# Editorial: Antioxidants in mitigating oxidative stress-induced damage

**DOI:** 10.3389/fcell.2025.1756697

**Published:** 2025-12-12

**Authors:** Marisol Fernandez-Ortiz, Ramy K. A. Sayed, Russel J. Reiter

**Affiliations:** 1 Greehey Children's Cancer Research Institute, University of Texas Health Science Center, San Antonio, TX, United States; 2 Department of Anatomy and Embryology, Faculty of Veterinary Medicine, Sohag University, Sohag, Egypt; 3 Department of Cellular and Structural Biology, University of Texas Health Science Center, San Antonio, TX, United States

**Keywords:** antioxidant, damage, oxidative stress, redox, reactive oxygen species (ROS), signaling

Oxidative stress arises from an imbalance between the generation of reactive oxygen species (ROS) and the antioxidant defenses that normally maintain redox homeostasis. When ROS accumulate in excess, they damage lipids, proteins, and nucleic acids, impair mitochondrial function, disrupt signaling networks, and compromise cell viability. Because this redox imbalance contributes to aging and to the pathophysiology of neurodegeneration, organ toxicity, reproductive decline, cancer, and metabolic disorders, there is a pressing need to understand how antioxidant strategies can counteract ROS-induced injury. The Research Topic *Antioxidants in Mitigating Oxidative Stress-Induced Damage* was established to gather studies that illuminate mechanisms of oxidative damage and explore the protective effects of antioxidants across diverse biological systems. With the full set of contributions now published, this Editorial highlight central findings, thematic insights, and future directions that emerge from this Research Topic.

The seven articles included in this Research Topic illustrate the breadth of contexts in which oxidative stress shapes disease processes and the variety of antioxidant-based strategies capable of mitigating such effects. Several studies focus on neuroprotection in toxin-induced models of Parkinson’s disease. Work on harpagoside demonstrates that this natural antioxidant reduces nitration of α-synuclein, thereby protecting neuronal cells challenged with rotenone. In a complementary study, paederoside also confers cytoprotection in rotenone-induced models, further underscoring the potential of naturally derived compounds to temper oxidative stress in neurodegenerative pathways.

Organ toxicity represents another domain where oxidative stress exerts far-reaching effects, and the issue includes an investigation into how green-synthesized silver nanoparticles produced from edible plant extracts can ameliorate cadmium chloride-induced hepatorenal and testicular injury in rats. These nanoparticles, endowed with intrinsic antioxidant properties, attenuate heavy-metal-mediated oxidative damage and point to innovative strategies for counteracting environmental toxicants. Their protective capacity highlights both the promise of nanoparticle-based approaches and the need for careful evaluation of safety and long-term effects as such technologies move toward translational consideration.

The role of oxidative stress in reproductive aging is addressed in a study examining placental trophoblasts from individuals of advanced maternal age. This work reveals increased oxidative damage accompanied by decreased YAP signaling, suggesting that redox imbalance contributes to placental aging and may underlie adverse maternal-fetal outcomes. By linking oxidative injury to regulatory pathways in trophoblast function, the findings emphasize the importance of antioxidant mechanisms not only in protecting cellular integrity but also in preserving physiological function across reproductive lifespan.

Another contribution explores the intricate interplay between ROS and ERK activation in cervical cancer cells. Rather than serving solely as damaging agents, ROS in this context modulate key signaling pathways that influence proliferation and survival. This study reinforces the concept that redox biology extends beyond macromolecular injury and can actively reshape cell behavior through redox-sensitive signaling cascades. For cancer research, this insight highlights the dual nature of oxidative stress-capable of inflicting harm but also of driving oncogenic processes-thereby influencing how antioxidant strategies might be applied in therapeutic settings.

Translational potential is further highlighted in a comprehensive review evaluating the therapeutic prospects of natural antioxidants for diabetic neuropathy. This work synthesizes evidence from experimental and clinical studies showing that antioxidant compounds can alleviate oxidative stress-mediated nerve damage in diabetes. While promising, the review also emphasizes challenges that must be addressed before antioxidant therapies can be fully integrated into clinical care, including issues of bioavailability, dosing, and long-term efficacy.

The Research Topic also includes a manuscript about endogenous antioxidant modulation under UV-induced stress. By examining the roles of CLIC1 and CLIC4, the study identifies cellular proteins that help confer resistance against UV-mediated oxidative injury. These findings demonstrate that intrinsic protective mechanisms can be strengthened or harnessed, potentially informing strategies for enhancing cellular defense in tissues frequently exposed to environmental stressors.

Across these diverse lines of research, several unifying themes emerge. First, antioxidant interventions demonstrate protective effects across multiple systems, stressors, and biological models. Whether in the context of neurotoxins, heavy metals, UV exposure, metabolic disease, or reproductive aging, antioxidant agents (ranging from natural compounds and biosynthesized nanoparticles to endogenous proteins) consistently reduce oxidative damage and help restore cellular function. Second, oxidative stress impacts more than the structural integrity of macromolecules. It reshapes signaling pathways, alters gene expression, and influences cellular decisions related to survival, proliferation, and differentiation. This broader influence underscores why antioxidant research must extend beyond measuring ROS levels to understanding how redox imbalance affects cellular networks at multiple biological scales.

The variety of experimental systems represented, spanning *in vitro* models, animal studies, mechanistic work, and translational reviews, demonstrates the importance of using diverse approaches to capture the complexity of redox biology. Mechanistic insights gained from cellular models can be validated in more physiologically relevant organisms, helping to identify therapeutic opportunities and refine potential interventions. Together, these studies suggest that while antioxidant-based strategies hold genuine translational promise, particularly for conditions such as diabetic neuropathy and heavy-metal toxicity, their development must proceed carefully. Considerations such as delivery strategies, safety profiles, tissue targeting, and optimal dosing remain central challenges to be addressed.

Looking to the future, several priorities emerge for advancing antioxidant research. Deeper mechanistic work is needed to clarify how antioxidants regulate redox-sensitive signaling pathways, mitochondrial dynamics, and stress responses. Improving delivery strategies, such as nanoparticle carriers, prodrugs, or engineered delivery systems, will be essential for translating promising compounds into effective therapies. Continued *in vivo* validation and well-designed clinical studies will help determine which antioxidant strategies are most viable for treating neurodegeneration, metabolic disease, reproductive aging, and organ toxicity. Finally, future research should aim not to eliminate ROS wholesale, given their physiological roles, but rather to restore redox balance in a context-appropriate manner.

In conclusion, the contributions to *Antioxidants in Mitigating Oxidative Stress-Induced Damage* collectively deepen our understanding of how oxidative stress affects diverse biological systems, and illustrate the breadth of antioxidant strategies capable of counteracting these effects. From cellular defense mechanisms and neuroprotection, to organ toxicity mitigation, reproductive health, cancer signaling, and metabolic disease, this Research Topic underscores the continued relevance and promise of antioxidant research. As Topic Editors, we believe this Research Topic provides a solid foundation for future investigations, translational efforts, and collaborative research aimed at harnessing antioxidants to improve health outcomes across diseases linked to oxidative stress. We thank all contributing authors and reviewers for their valuable work and dedication.

